# Do pregnancy outcomes of women with false-positive early gestational diabetes mellitus differ from those of women with normal glucose tolerance?

**DOI:** 10.1186/s12902-022-01124-1

**Published:** 2022-08-13

**Authors:** Sayuri Nakanishi, Shigeru Aoki, Ryosuke Shindo, Soichiro Obata, Junko Kasai, Etsuko Miyagi

**Affiliations:** 1 Perinatal Center for Maternity and Neonates, Yokohama City University Medical Center, 4-57 Urafunecho, Minami-ku, Yokohama City, Kanagawa 232-0024 Japan; 2 Department of Obstetrics and Gynecology, Yokohama City University School of Medicine, 3-9 Fukuura, Kanazawa-ku, Yokohama City, , Kanagawa 236-0004 Japan

**Keywords:** Gestational diabetes mellitus, Early-onset gestational diabetes mellitus, Pregnancy outcomes

## Abstract

**Background:**

To investigate whether false-positive early gestational diabetes mellitus (GDM) women can be managed similarly as normal glucose tolerance (NGT) women.

**Methods:**

This retrospective study was conducted at a tertiary care center in Japan. Pregnancy and neonatal outcomes of 67 singleton pregnancies with false-positive early GDM and 1774 singleton pregnancies with NGT who delivered after 22 weeks of gestation were compared. GDM was diagnosed according to the International Association of Diabetes and Pregnancy Study Groups (IADPSG) criteria (patients having one or more of the following: fasting plasma glucose ≥ 92 mg/dL and a 75 g oral glucose tolerance test (OGTT) value ≥ 180 mg/dL at 1 h, or ≥ 153 mg/dL at 2 h). Pregnant women diagnosed with GDM in early pregnancy who did not meet the diagnostic criteria on the second OGTT were defined as having false-positive early GDM. Women with false-positive early GDM did not receive any therapeutic intervention during gestation.

**Results:**

Maternal age, pre-pregnancy body mass index, and gestational weight gain were significantly higher in the false-positive GDM group than in the NGT group. No significant differences were found in pregnancy outcomes, including gestational age, birth weight, large for gestational age rate, and cesarean delivery rate. Except for a higher neonatal hypoglycemia rate in the false-positive early GDM group, no significant differences were found in neonatal outcomes.

**Conclusions:**

There were no clinically significant differences between early GDM false-positive women exhibiting GDM patterns only during early pregnancy and NGT women. False-positive early GDM women can be managed similarly as NGT women, suggesting that World Health Organization diagnostic guidelines, applying the IADPSG criteria during early pregnancy, need revision.

## Background

Since the diagnosis and treatment of gestational diabetes mellitus (GDM) improves pregnancy outcomes [1, 2], screening for GDM in pregnancy is an important treatment practice. In 2010, the International Association of Diabetes and Pregnancy Study Groups (IADPSG) indicated that a diagnosis of GDM should be made if one or more of the following results are obtained from the 75 g oral glucose tolerance test (OGTT) conducted between 24 and 28 weeks of gestation: 92 mg/dL for a fasting plasma glucose (FPG) concentration, 180 mg/dL for a 1 h postprandial plasma glucose (1 h PG) concentration, or 153 mg/dL for 2 h postprandial plasma glucose (2 h PG) concentration [3]. In 2010, physicians were also advised to make a diagnosis of GDM if an FPG of 92 mg/dL was observed in early pregnancy. In 2016, this recommendation concerning early pregnancy was retracted, and the IADPSG recommended that early-onset GDM be considered if an HbA1c level of 5.9% or higher is observed in early pregnancy [4]. However, since 2013, the World Health Organization (WHO) has recommended that if any of the IADPSG threshold values are met at any time during pregnancy, GDM should be diagnosed [5]. In Japan, the WHO diagnostic criteria for GDM have been employed since 2010 [6].

However, no conclusive evidence has yet been found suggesting that diagnosis and treatment of GDM in early pregnancy improves pregnancy outcomes [7–9]. Thus, to investigate the clinical significance of early-onset GDM, we conducted the Timing of Therapeutic Intervention for Gestational Diabetes Mellitus (TTIGDM) study, which was designed to evaluate the validity and efficacy of treating GDM (detected in early pregnancy) during mid-pregnancy. In the TTIGDM study, we monitored pregnant women diagnosed with GDM in early pregnancy according to the IADPSG criteria without performing clinical interventions until mid-pregnancy. The women received a 75 g OGTT by 20 weeks of gestation and again during 24–28 weeks of gestation—the IADPSG-recommended testing period. We diagnosed only those women who exhibited a GDM pattern at this time as having true GDM and treated them as patients with GDM accordingly. We found that if early-onset GDM was left untreated and simply monitored, approximately half of all patients did not exhibit a GDM pattern with their second OGTT during mid-pregnancy. Such women with apparent GDM in early pregnancy but apparent normal glucose tolerance (NGT) in mid-pregnancy were classified as “false-positive early GDM” [10].

However, false-positive early GDM was the concept that we first reported in a previous report(10), it remains unclear whether women exhibiting a false-positive early GDM pattern should be considered as having impaired glucose tolerance. Thus, in the present study, we compared the pregnancy outcomes of women with false-positive early GDM to those of women with NGT to investigate whether false-positive early GDM women can indeed be managed in the same way as NGT women.

## Methods

### Study design

Sixty-seven pregnant women with false-positive early GDM were selected from the TTIGDM study, and 1774 pregnant women with NGT were selected among pregnant women who delivered neonates after 22 weeks of gestation. These populations were then compared. The protocol for this research project has been approved by a suitably constituted Ethics Committee of the institution and it conforms to the provisions of the Declaration of Helsinki. Committee of the Yokohama City University Medical Center, Approval No. F211000053.

### Participants

The participants of this study consisted of 67 individuals selected from the TTIGDM study with false-positive early GDM, as defined below, who delivered single neonates after 22 weeks of gestation at the Yokohama City University Medical Center. The TTIGDM study was a prospective cohort trial conducted between January 2018 and December 2019 at five secondary and tertiary medical facilities in Japan. Among patients with high-risk of GDM, we targeted pregnant women who underwent a 75 g OGTT prior to 20 weeks of gestation and diagnosed women with GDM if they exceeded IADPSG thresholds defined by one or more of the following: FPG level ≥ 92 mg/dL, 1 h post-prandial glucose ≥ 180 mg/dL, and/or 2 h post-prandial glucose ≥ 153 mg/dL. The definition of high-risk of GDM has previously been described in detail [10].

**Fig. 1:**
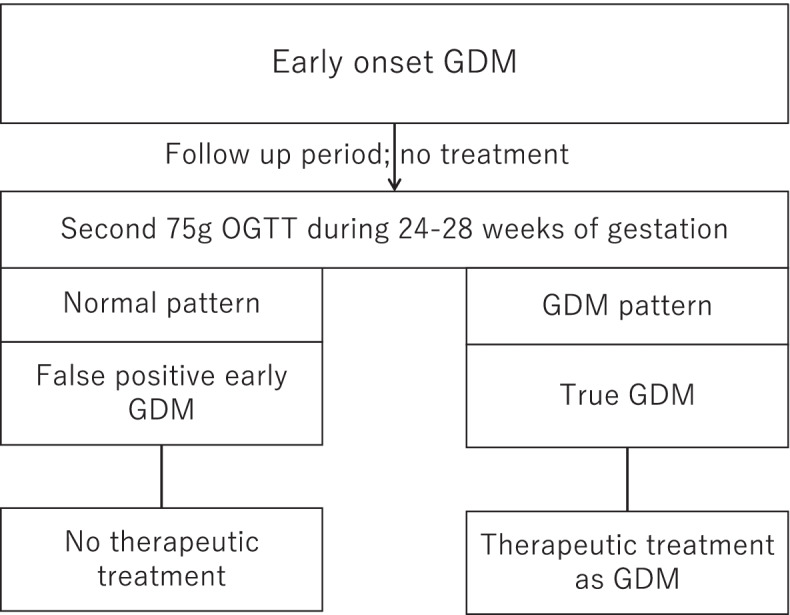
Timing of Therapeutic Intervention for Gestational Diabetes Mellitus study design. Abbreviations: GDM, gestational diabetes mellitus; OGTT, oral glucose tolerance test

As shown in Fig. 1, the enrolled study participants did not receive any therapeutic intervention until the repetition of the 75 g OGTT at 24–28 weeks of gestation. Pregnant women who met the IADPSG criteria at 24–28 weeks gestation were designated as true GDM patients and initiated therapy. Women diagnosed with GDM in early pregnancy who did not meet the diagnostic criteria during the second OGTT were defined as having false-positive early GDM. Women with false-positive early GDM did not receive any therapeutic intervention during gestation.

### Control

The control group comprised 1774 pregnant women diagnosed with NGT by screening process performed during early and mid-pregnancy and who delivered single neonates after 22 weeks of gestation between January 2018 and December 2019 at the Yokohama City University Medical Center. In the GDM screening process, in early pregnancy, a 75 g OGTT was performed if the random blood glucose result was ≥ 95 mg/dL during early pregnancy and/or if pregnant women were at high risk of GDM; in mid-pregnancy, a 50 g GCT was provided to all women who were not diagnosed with GDM in early pregnancy, and a 75 g OGTT was performed if a 50 g glucose challenge test (GCT) result was ≥ 140 mg/dl. NGT women were defined as those who did not have a final diagnosis of GDM as per IADPSG thresholds in the screenings during pregnancy.

### Study outcomes

We examined maternal characteristics, such as maternal age, pre-pregnancy BMI, and nulliparity rate (%). Pregnancy outcomes included gestational age (weeks), cesarean delivery rate, emergency cesarean delivery rate, gestational weight gain (GWG), birth weight (g), large for gestational age (LGA) rate, small for gestational age (SGA) rate, and rates of hypertensive disorders of pregnancy (HDP). We examined pregnancy and neonatal outcomes, such as the neonatal intensive care unit (NICU) admission rate, neonatal hypoglycemia, respiratory distress syndrome (RDS), and neonatal hyperbilirubinemia, using both univariate and multivariate analyses.

The amount of GWG was defined as the difference between the body weight at delivery and pre-pregnancy body weight. LGA neonates were defined as those with a birth weight ≥ 90^th^ percentile. On the other hand, SGA neonates were defined as those with birth weights < 10^th^ percentile. Neonatal hypoglycemia was defined as a blood glucose level of < 40 mg/dL. In the false-positive early GDM group, blood glucose level was measured in all neonates after birth. In the NGT group, neonatal blood glucose level was measured only if neonates were SGA or LGA, if NICU admission was required, or if judged necessary by the neonatologist. The presence or absence of hyperbilirubinemia, defined as the need for phototherapy in neonates, was confirmed in all patients of both groups.

RDS was defined by characteristic findings on chest radiographic examination and oxygen requirement within 24 h after birth. Macrosomia was defined as a birth weight exceeding 4000 g. Shoulder dystocia was defined clinically as the presence of difficulty in delivering the shoulder after delivery of the neonate's head and the necessity for some form of treatment or procedure.

### Statistical analyses

JMP pro 15 (SAS Institute Inc., North Carolina, USA) was used for statistical analyses. The medians and proportions were compared using the Mann–Whitney U-test and Fisher’s exact test, respectively. The level of statistical significance (p) was set at < 0.05. In multivariate analyses, the adjusted regression coefficient (aRC), adjusted odds ratio (aOR), and 95% confidence intervals (CIs) were used.

## Results

Maternal characteristics are shown in Table 1. The median maternal age in the false-positive early GDM group was significantly higher than that of the NGT group (37 vs. 33 years, *p* < 0.001). Pre-pregnancy BMI was also significantly higher in the false-positive early GDM group than in the NGT group (22.2 kg/m^2^ vs. 20.5 kg/m^2^; *p* = 0.005).

**Table 1:** Maternal characteristics

	Normal glucose tolerance group *n* = 1774	False-positive early GDM group *n* = 67	*p*-value
Age (years)	33 [29–36]	37 [34–39]	< 0.001
Pre-pregnancy BMI (kg/m^2^)	20.5 [19.1–22.6]	22.2 [19.3–25.1]	0.005
Primipara	898 (51%)	26 (39%)	0.62

*GDM* Gestational diabetes mellitus, *BMI* Body mass index

Values are expressed as median [IQR] or n (%)

Pregnancy outcomes are shown in Table 2. No significant differences between groups were observed as regards gestational age or cesarean delivery rate. The false-positive early GDM group had significantly less GWG than the NGT group (8.4 kg vs. 10.3 kg, *p* = 0.003). The same result was obtained using multivariate analysis (aRC − 0.681, 95% CI [− 1.200 to − 0.162]). No significant difference was observed in the LGA rate between the NGT (9%) and false-positive early GDM groups (9%) via either univariate or multivariate analysis (*p* = 1.000, aOR 0.868, 95% CI [0.361–2.087]).

**Table 2:** Pregnancy and neonatal outcomes

	Normal glucose tolerance group *n* = 1774	False-positive early GDM group *n* = 67	*p*-value	aRC or aOR	95%CI
Gestational age (weeks)	39.2 [38.2–40.1]	39.2 [38.2–40.1]	0.41	0.273	-0.289–4.118
Birth weight (g)	2982 [2700–3233]	3088 [2778–3312]	0.15	46.88	-20.463–114.236
LGA	159 (9.0%)	6 (9.0%)	1.00	0.868	0.361–2.087
SGA	168 (9.5%)	4 (6.0%)	0.20	0.460	0.142–1.497
Macrosomia	11 (0.62%)	0 (0%)			
Shoulder dystocia	13 (0.73%)	0 (0%)			
Gestational weight gain (kg)	10.3 [7.7–12.7]	8.4 [4.6–11.8]	0.003	-0.681	-1.200- -0.162
HDP	140 (7.9%)	12 (18%)	0.003	1.34	0.834–3.277
Cesarean delivery	470 (26%)	24 (36%)	0.093	1.023	0.603–1.738
Emergency cesarean delivery	221 (13%)	12 (18%)	0.19	1.134	0.589–2.182
NICU admission	296 (17%)	10 (15%)	0.74	0.782	0.370–1.567
Neonatal hypoglycemia^a^	10 (0.5%)	4 (6.0%)	0.001	11.3	3.125–41.407
RDS	22 (1.2%)	0(0%)			
Neonatal hyperbilirubinemia	174 (9.8%)	8 (12%)	0.53	1.175	0.544–2.535

Values are expressed as median [IQR] or n (%). Moreover, aOR and aRC have been adjusted for age and pre-pregnant BMI

*GDM* Gestational diabetes mellitus, *LGA* Large for gestational age, *SGA* Small for gestational age, *HDP* Hypertensive disorder of pregnancy, *NICU* Neonatal intensive care unit, *RDS* Respiratory distress syndrome, *aOR* Adjusted Odds ratio, *aRC* Adjusted Regression Coefficient

^a^In the false-positive early GDM group, blood glucose level was measured in all neonates after birth. In the NGT group, the blood glucose level of neonates was only measured if neonates were SGA or LGA, if NICU admission was required, or if judged necessary by the neonatologist

Macrosomia and shoulder dystocia did not occur in the false-positive early GDM group. No significant differences were observed between the groups in terms of NICU admission, or neonatal hyperbilirubinemia. However, neonatal hypoglycemia was more common in the false-positive early GDM group via either univariate or multivariate analysis (*p* = 0.001, aOR 11.3, 95% CI [3.125–41.407]).

## Discussion

Between the false-positive early GDM which showed impaired glucose tolerance only in early pregnancy and NGT which showed no impaired glucose tolerance in early and mid pregnancy, no significant differences in pregnancy outcomes such as LGA rates and cesarean delivery. However, neonatal hypoglycemia rate was higher in false-positive early GDM.

There is no consensus on the screening and intervention for GDM in early pregnancy [11–13], and a systematic review of early-onset GDM also stated that any benefit or possible harm caused by the treatment of early-onset GDM should be investigated as soon as possible [7]. In recent years, several randomized controlled trials (RCTs) on impaired glucose tolerance in early pregnancy have been reported. Harper et al. [14] selected obese women, who are at high risk of GDM, and used a two-step screening method involving a 50 g GCT and a 100 g OGTT to conduct an RCT, wherein patients were divided into either a group in which GDM screening was carried out until 20 weeks of gestation or another group in which the normal GDM screening protocol (at 24–28 weeks of gestation) was carried out. No significant differences in composite primary outcomes, including macrosomia, cesarean delivery, HDP, hyperbilirubinemia, shoulder dystocia, and neonatal hypoglycemia, were observed between the groups screened early during pregnancy and the group screened at 24–28 weeks of gestation. Even after limiting their analysis to only those cases diagnosed with GDM, no significant differences were found in composite primary outcomes between groups, and it was concluded that screening for GDM in early pregnancy did not improve pregnancy outcomes even in obese women. Roeder et al. [15] carried out an RCT including pregnant women who exhibited early pregnancy hyperglycemia, defined as high fasting blood glucose or an HbA1c of 5.7% or higher in early pregnancy. They examined differences in outcomes between a group that received first trimester therapeutic interventions and a group that received third trimester therapeutic interventions. No significant differences were observed in the rates of macrosomia or of use of pharmacotherapies for blood glucose control between the groups. Although this RCT was underpowered, it indicated that early therapeutic interventions produced no discernible effects even among pregnant women with hyperglycemia in early pregnancy.

Liu et al. [16] limited themselves to low-risk pregnant women in a prospective cohort study in which a 75 g OGTT was performed twice during pregnancy. No significant differences, whether by univariate or multivariate analyses, were noted in LGA, macrosomia, neonatal hyperinsulinemia, neonatal hypoglycemia, or pregnancy-induced hypertension in women who exhibited a GDM pattern only in early pregnancy compared to women with NGT during both early and mid-pregnancy. Our study was similar to that of Liu et al. [16], which was limited to low-risk pregnant women, except for our finding that neonatal hypoglycemia was more common in the false-positive early GDM group.

Similar to the findings of Harper et al. [14] and Roeder et al. [15], who both reported that even among pregnant women at high risk for GDM, they found no benefits of therapeutic intervention from early pregnancy for mild glucose intolerance in early pregnancy, our findings suggest that a 75 g OGTT conducted in early pregnancy without any follow-up therapeutic interventions would not significantly affect pregnancy outcomes, even in women with risk factors for GDM.

In contrast, we observed that neonatal hypoglycemia was significantly higher in the false-positive early GDM group. It has been reported that even in healthy term infants, transient hypoglycemia of < 47 mg/dl is observed in approximately 40% of cases when blood glucose level monitoring is continued for 5 days after birth [17]. In our control (NGT) group, blood glucose was measured only in cases wherein the neonate was judged to be at risk of neonatal hypoglycemia. Therefore, we presumed that some newborn babies were hypoglycemic but were overlooked due to a lack of symptoms. As a result, this may have led to a higher incidence of neonatal hypoglycemia in the false-positive early GDM group, wherein examinations were carried out in all cases.

Our study has several limitations. First, the results may not be applicable to other races, since all participants were Japanese. Second, the number of cases was small, and it is possible that a β error occurred. Third, since the subjects of the false-positive early GDM group were aware that they would normally have been diagnosed with GDM in Japan, it is possible that this knowledge caused some lifestyle-related changes. In fact, the false-positive early GDM group had significantly less GWG than the NGT group. The possibility that this may have affected pregnancy outcomes cannot be ruled out. Contrarily, to the best of our knowledge, our study is the first to demonstrate that pregnancy outcomes do not significantly differ between false-positive early GDM pregnant women selected from women with risk factors for GDM and NGT women.

## Conclusion

In conclusion, pregnant women with false-positive early GDM can be managed similar to patients with NGT. Moreover, there is little clinical significance to patients with GDM exhibiting GDM patterns only during early pregnancy. Therefore, the WHO diagnostic guidelines, which apply the IADPSG criteria during early pregnancy, should be revised.

## Data Availability

The datasets generated and/or analyzed during the current study are not publicly available due to limitations of ethical approval involving the patient data and anonymity but are available from the corresponding author on reasonable request.

## Abbreviations

GDM: Gestational diabetes mellitus
NGT: Normal glucose tolerance
OGTT: Oral glucose tolerance test
IADPSG: The International Association of Diabetes and Pregnancy Study Groups
FPG: Fasting plasma glucose
1hPG: 1 H postprandial plasma glucose
2hPG: 2 H postprandial plasma glucose
TTIGDM study: Timing of Therapeutic Intervention for Gestational Diabetes Mellitus study
GCT: Glucose challenge test
GWG: Gestational weight gain
LGA: Large for gestational age
SGA: Small for gestational age
HDP: Hypertensive disorders of pregnancy
NICU: Neonatal intensive care unit
RDS: Respiratory distress syndrome
aRC: The adjusted regression coefficient
aOR: Adjusted odds ratio
CIs: Confidence intervals
RCT: Randomized controlled trial
